# Increased lipid availability for three days reduces whole body glucose uptake, impairs muscle mitochondrial function and initiates opposing effects on PGC-1α promoter methylation in healthy subjects

**DOI:** 10.1371/journal.pone.0188208

**Published:** 2017-12-20

**Authors:** Roy Eldor, Luke Norton, Marcel Fourcaudot, Cynthia Galindo, Ralph A. DeFronzo, Muhammad Abdul-Ghani

**Affiliations:** 1 Diabetes Unit, Institute for Metabolism, Endocrinology and Hypertension, Tel Aviv Sourasky Medical Center, Tel Aviv, Israel; 2 Division of Diabetes, University of Texas Health Science Center, San Antonio, Texas, United States of America; Virgen Macarena University Hospital, School of Medicine, University of Seville, SPAIN

## Abstract

**Aims:**

FFA and FFA metabolites cause insulin resistance and impair beta cell function. The goal of our research was to examine whether elevation of plasma FFA impairs mitochondrial function and alters PGC-1α promoter methylation.

**Methods:**

In this uncontrolled, change from baseline study design, insulin sensitivity and glucose-stimulated insulin secretion were measured in 9 normal glucose tolerant subjects before and after 3 day lipid infusion to elevate plasma FFA concentration. Vastus lateralis muscle biopsies were obtained and mitochondrial function, PGC-1α expression, and PGC-1α promoter methylation were quantitated.

**Results:**

Increased plasma FFA (440±93 μmol/Lto 997±242 μM, *p*<0.001) decreased insulin-stimulated total glucose disposal (TGD) by 25% (*p* = 0.008), impaired suppression of endogenous glucose production (*p* = 0.01), and reduced mitochondrial ATP synthesis with complex 1 (34%, *p*<0.05) and complex 2 (30%, *p*<0.05) substrates. Lipid infusion had no effect on muscle PGC-1α RNA expression, total methylation or non-CpG methylation, but methylation of the alternative PGC-1α promoter decreased (1.30±0.30 to 0.84±0.15% methylated residues/patient•strand, *p* = 0.055). Within PGC-1α promoter there was demethylation of CpT residues (0.72±0.16 vs. 0.28±0.10 methylated residues/patient•strand) (*p* = 0.002), which was inversely correlated with PGC-1α mRNA expression (r = -0.94, *p*<0.0001) and ATP synthesis with complex 1 (r = -0.80, p<0.01) and complex 2 (r = -0.69, *p*<0.05) substrates. Lipid infusion increased DNMT-3B (methyltransferase associated with PGC-1α promoter non-CpG methylation) mRNA expression (0.87 ± 0.09 to 1.62 ± 0.22 arbitrary units, *p* = 0.005), which correlated inversely with CpT demethylation (r = 0.67, *p*<0.05).

**Conclusion/Interpretation:**

Physiologic plasma FFA elevation in NGT individuals has opposing effects on PGC-1α non-CpG residue methylation (CpT demethylation and increased DNMT-3B expression), which is correlated with changes in PGC-1α expression and skeletal muscle mitochondrial function.

## Introduction

Insulin resistance is a key pathophysiologic derangement in type 2 diabetes mellitus (T2DM) and is strongly associated with obesity [1]. Lipotoxicity plays a pivotal role in development of insulin resistance [2–4]. Studies from our laboratory and others have shown that acute exposure (2–6 hour) to supraphysiological plasma free fatty acid (FFA) levels (increased to 5–10 fold above fasting levels) reduced insulin sensitivity in healthy individuals and in individuals with T2DM [5–9]. However, the clinical relevance of these experiments was limited since FFA levels in healthy individual under normal living conditions rarely rise to such high levels. A more clinically relevant approach is to increase the plasma FFA level to the high physiologic range as seen in T2DM and obese individuals [9]) for longer a period of time (2–4 days). We have shown that this results in both an increase in insulin resistance in healthy individuals [10] and impaired insulin secretion in genetically predisposed, normal-glucose-tolerant (NGT) individuals[11]. Conversely, sustained plasma FFA reduction with acipimox in NGT subjects with strong family history of T2DM and in obese NGT and T2DM subjects enhances insulin-mediated muscle glucose disposal and suppression of hepatic glucose production [12, 13].

*In vivo* and *ex vivo* studies [14–16] have demonstrated impaired mitochondrial function in skeletal muscle of insulin resistant individuals. We have shown that reduction in plasma FFA with acipimox improves mitochondrial ATP synthesis rate by >50% (7). The increase in ATP synthesis rate correlated closely with the decrease in plasma FFA and increase in insulin-mediated glucose disposal.

A potential link between lipotoxicity and mitochondrial dysfunction may be peroxisome proliferator-activated receptor γ co-activator 1α (PGC-1α) which plays a central role in regulating skeletal muscle mitochondrial function and biogenesis in response to changes in plasma and intracellular lipid levels [17, 18]. We previously have shown that lipid infusion for 48- hours resulted in a decrease in PGC-1α mRNA expression [19]. Hypermethylation of the PGC-1α promoter was identified in a genome-wide analysis screening for differential promoter DNA methylation in T2DM [20]. PGC-1α promoter methylation has been implicated in development of mitochondrial dysfunction following an increase in plasma/intracellular lipid levels. In low birth weight subjects, high-fat overfeeding caused peripheral insulin resistance, reduced PGC-1α gene expression, and increased PGC-1α promoter methylation at sequence CpG sites. However, PGC-1α promoter methylation did not correlate with PGC-1α mRNA expression [21]. In skeletal muscle of T2DM individuals increased methylation of non-CpG nucleotides of PCG-1α promoter was negatively correlated with PGC-1α mRNA expression and responsive to acute *ex vivo* FFA exposure, but not to insulin [20].

We hypothesized that mitochondrial dysfunction could be induced in healthy NGT individuals by short-term increased lipid availability and that PGC-1α promoter methylation played a pivotal role in mediating this negative interaction. The aim of our research was to examine whether elevation of plasma FFA impairs mitochondrial function and alters PGC-1α promoter methylation.

## Methods

### Subjects

Eleven subjects were recruited between July 2007 and February 2009. Two subjects withdrew consent during the study and nine healthy normal glucose tolerant (NGT) subjects [4 females/5 males; age = 37.2±3.2 years (range 22–40 years); BMI = 23.6 ± 1.2 kg/m2; FPG = 99±3 mg/dl] completed the study. All subjects had a normal 2-hour OGTT. Body weight was stable (±2 lbs) over 3 months prior to study and no subject participated in an excessively heavy exercise program. Routine screening blood tests, urinalysis, thyroid function, and EKG were normal. Protocol was approved by Institutional Review Board of University of Texas Health Science Center at San Antonio and informed written consent was obtained before participation.

### Study design

After screening, eligible subjects received: (i) 4-hour euglycemic insulin clamp (80 mU/m^2^min) with vastus lateralis muscle biopsies before the start of insulin clamp, (ii) 2-step hyperglycemic clamp (+100 and +300 mg/dl), (iii) DEXA scan. After completing baseline studies, subjects were admitted to the CRC and received intravenous 20% neutral triglyceride solution (Liposyn III; Hospira Inc., Lake Forest, IL) containing 54.5% linoleic, 22.4% oleic, 10.5% palmitic, 4.2% stearic, and 8.3% linolenic acid (1 ml/minute) for 4 days and heparin (0.2 units per kilogram per min) on days 1 and 2 [22]. On days 3 and 4, euglycemic insulin and hyperglycemic clamp studies [23] were repeated.

### OGTT

A catheter was placed into an antecubital vein and blood samples were collected at –30, –15, 0, 30, 60, 90 and 120 min for measurement of plasma glucose, insulin, C-peptide and FFA.

### Dual X-ray absorptiometry

Dual X-ray absorptiometry (Hologic Inc., Waltham, MA) was performed to measure fat and fat-free mass (FFM).

### Euglycemic insulin clamp [23]

Following 10-hour overnight fast, a catheter was placed into antecubital vein for infusion of all test substances. A second catheter was inserted retrogradely into vein on dorsum of hand which was placed into a thermoregulated box heated to 70°C. At 0800 hours, participants received prime (40 μCi × fasting plasma glucose/90)–continuous (0.40 μCi/min) infusion of [3-^3^H] glucose (DuPont NEN Life Science, Boston, MA). After 2-hour basal tracer equilibration period, participants received prime–continuous insulin infusion at 240 pmol•min^−1^•m^−2^ (80 mU•min^−1^•m^−2^) for 240 min. During last 30 min of basal equilibration period, plasma samples were taken at 5–10 min intervals for determination of plasma glucose and insulin concentrations and [^3^H] glucose radioactivity. During insulin infusion, plasma glucose was measured every 5 minutes, and variable infusion of 20% glucose was adjusted to maintain plasma glucose at each participant’s fasting level with coefficient of variation <5%. Plasma samples were collected every 5–15 min for determination of plasma glucose and insulin concentrations and [^3^H] glucose radioactivity.

### Muscle biopsies

Sixty minutes before insulin clamp, percutaneous vastus lateralis muscle biopsy (∼300 mg) was obtained [24]. Muscle biopsy was placed in buffer on ice for mitochondrial isolation and measurement of mitochondrial ATP synthesis and ROS production rate.

### Hyperglycemic clamp [23]

Following 10-hour overnight fast, a catheter was placed into antecubital vein for infusion of all test substances. A second catheter was inserted retrogradely into vein on dorsum of hand, and hand was placed into thermoregulated box heated to 70°C. After obtaining three baseline samples, plasma glucose was rapidly raised and maintained by +100 mg/dL above fasting for 120 minutes and then by +300 mg/dL for an additional 90 minutes with variable infusion of 20% glucose. At 210 minutes, a 5 gram bolus of arginine was infused over 1 minute while maintaining plasma glucose constant for another 30 minutes. Blood samples were collected at -30,−20, −10, 0, 2, 4, 6, 8, 10, 12, 15, 30, 45, 60, 75, 90, 100,110, 120, 122, 124, 126, 128, 130, 135, 150, 165, 180, 190, 200, 210, 212, 214, 216, 218, 220, 225, 230 and 240 minutes for measurement of plasma insulin and C-peptide concentrations.

### Analytical techniques

Plasma glucose was measured by glucose oxidase reaction (Glucose Oxidase Analyzer; Beckman, Fullerton, CA). Plasma insulin was measured by radioimmunoassay (Coat A Count; Diagnostic Products, Los Angeles, CA). Tritiated glucose-specific activity was determined on deproteinized barium/zinc plasma samples. Plasma FFA was measured by enzymatic colorimetric method (Wako Chemicals, Neuss, Germany).

### Mitochondrial purification

Mitochondria were purified from muscle tissue as previously described [25]. Mitochondrial integrity was assessed by respiratory control ratio (>6 with pyruvate) at end of each experiment. All procedures were performed on ice and entire isolation procedure lasted ~ 60 min. Final mitochondrial solution was kept on ice and used immediately following isolation.

### Mitochondrial ATP production

Mitochondrial ATP synthesis rate was measured *ex vivo* with chemiluminescence technique as previously described [13]. Briefly, mitochondria were isolated from fresh muscle tissue by differential centrifugation. 4 μg of mitochondrial protein was aliquoted to each reaction well. Substrates were added as follows: 2.5 mM pyruvate, 2.5 mM glutamate, 5 mM succinate plus 0.001 mM rotenone, 0.5 mM palmitoyl-L-carnitine. 2.5 mM malate was added to complex I substrates. Luciferine/luciferase was added to monitor ATP production. After 5 minute incubation at 37°C, substrates were added and reaction was started by addition of adenosine diphosphate (ADP) [25].

### Mitochondrial ROS production

The rate of mitochondrial ROS production was measured by quantitation of the release of mitochondrial H_2_O_2_ with the fluorescent dye Amplex Red (Molecular Probes, Eugene, OR) as previously described [14]. ROS production rate was performed in mitochondria under state II (with substrate and without the addition of ADP) conditions. The substrate concentrations were the same as with the measurement of ATP synthesis. Fluorescence was observed at 530 nm excitation and 590 nm emission for 5 min. The slope in fluorescence was converted to the H_2_O_2_ production rate using a standard curve [15].

### Bisulfite sequencing

Bisulfite treatment was performed using EpiTect Bisulfite Kit (QIAGEN) according to manufacturer’s protocol. DNA was extracted from a fragment of the vastus lateralis muscle obtained by biopsy. For amplification of region from 337 to 37 of PGC-1α promoter, the following primers were used: sense 50 TAT AGT TAT TTT GTT ATG AAA TAG GGA GTT TT G 30; antisense 50 CCA ATC ACA TAA CAA AAC TAT TAA AAA ATA A 30. For amplification of the region from 243 to 47 of the alternative PGC-1α promoter, the following primers were used: sense 50 ATA GGG TTG TTG GAA AGT ATA TGA TAT T 30; antisense 50 AAA AAA CAC TCA CAA CAA AAA CTT C 30. The obtained PCR fragments were purified from agarose gel using MinElute Gel Extraction Kit (QIAGEN) and cloned using pGEM^®^-T Easy vector system (Promega), according to manufacturer’s protocol. Individual clones were grown and plasmids purified using QIAprep Spin Miniprep Kit (QIAGEN) and EcoR1 Fast Digest restriction enzyme (Thermo Fisher Scientific, Rockford, IL). For each condition, 10–15 clones were sequenced using T7 promoter primer on ABI 3730xl DNA Analyzer platform (Cogenics, Hope End, UK).

### Quantitative PCR

Total RNA was isolated using TRIZOL reagent (Sigma-Aldrich, St Louis, MO). One-step RT-PCR was performed on ABI-Prism-7900HT System (Applied Biosystems, Foster City, CA). mRNA levels were normalized to HMBS. Primers were designed using Primer Express computer software (Applied Biosystems).

### Statistical analysis and calculations

Following an overnight fast, steady-state conditions prevail and endogenous glucose production (EGP) was calculated as [^3^H] glucose infusion rate (dpm/min) divided by steady state plasma [^3^H] glucose specific activity (dpm/mmol). During insulin clamp, non-steady-state conditions for [^3^H] glucose specific activity prevail, and rate of glucose appearance (R_a_) was calculated with Steele’s equation [26]. Rate of residual EGP during insulin clamp was calculated by subtracting exogenous glucose infusion rate from tracer-derived Ra. Insulin-stimulated total glucose disposal (TGD) rate was calculated by adding rate of residual EGP to exogenous glucose infusion rate.

ATP synthesis rate was measured as slope of chemi-luminescence over time. Slope of the blank well (mitochondria without substrate) was subtracted from slope obtained with any given substrate, and normalized per mg mitochondrial protein per min. Rate of H_2_O_2_ production was measured as slope of fluorescence over time. Slope of blank well was subtracted from slope obtained with any given substrate and normalized per mg mitochondrial protein per min. Methylation was calculated as mean of percent methylated cytosine residues from total and specific combinations of residues per patient.

Values are presented as mean ± standard error. For comparison of values obtained during insulin clamp performed with and without Liposyn infusion, ANOVA was used. Simple Pearson correlation was used to assess correlation between variables. Statistical analyses were performed with SPSS (version 14) (SPSS, Chicago, IL). Statistical significance was considered at *p*<0.05. Statistical significant differences were confirmed by Bonferroni test.

## Results

### Plasma FFA concentration

Baseline fasting plasma FFA concentration was 440±93 μmol/L. 72-hours of Liposyn infusion increased plasma FFA ~2-fold (997±242 μM, *p*<0.001).

### Insulin sensitivity in muscle and liver

**Fig 1:**
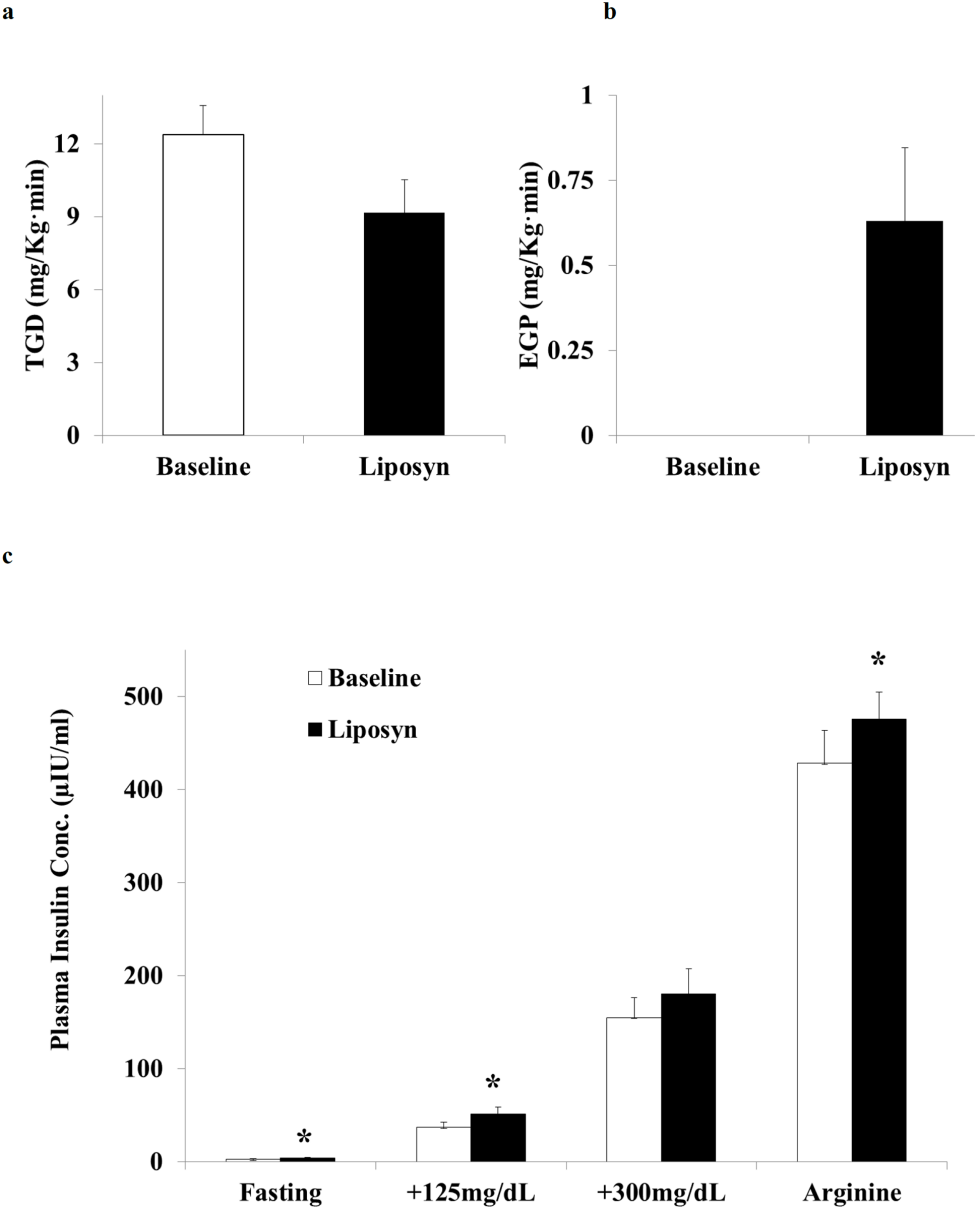
Effect of chronic (3 day) physiologic increase in plasma FFA concentration on insulin sensitivity parameters. **a:** insulin-stimulated total body glucose disposal (TGD) and **b**: suppression of endogenous glucose production (EGP) during the euglycemic insulin clamp. n = 9, *p<0.05. **c:** Plasma insulin concentration following an overnight fast and during the two-step hyperglycemic clamp (+125 and +300 mg/dL) with arginine. n = 9, *p<0.05.

3-day physiologic increase in plasma FFA increased basal fasting (pre-clamp) insulin levels from 3.6± 0.7 to 7.0 ± 0.9 mIU/ml (S1a Fig, *p*<0.001), reduced insulin-stimulated TGD from 12.1±1.0 to 9.2±1.2 mg/kg•min (*p* = 0.01) (Fig 1a), while insulin-induced suppression of endogenous glucose production was impaired (0.10±0.10 *vs*. 0.63±0.21 mg /kg•min *p* = 0.008) (Fig 1b and S1 Fig).

### Glucose-stimulated insulin secretion

The fasting plasma insulin concentration, as well as the plasma insulin response during all hyperglycemic clamp steps (+125mg/dL, +300 mg/dL and arginine), were higher after Liposyn infusion (but did not reach statistical significance during the +300 mg/dL step): fasting (2.3±0.7 *vs*. 4.0±0.7 μIU/ml, *p* = 0.03); +125mg/dL (time 90-120min: 37±5 *vs*. 51±7 μIU/ml; *p* = 0.01); +300mg/dL (time 90-120min: 154±21 *vs*. 180±26 *p* = 0.10); and after arginine (428±35 *vs*. 476±29 uU/ml; *p* = 0.01) (Fig 1c and S2 Fig), indicating the normal physiologic response to the insulin resistance associated with Liposyn infusion. The lack of statistical significance in insulin secretion in the 2^nd^ hyperglycemic clamp step may be due to the relatively small number of subjects in this study or perhaps to a specific mechanism by which extreme hyperglycemia may overcome the Liposyn mediated increase in glucose stimulated insulin secretion. We previously have shown in a similar study that prolonged Liposyn infusion to produce a physiologic increase in the plasma FFA concentration results in a significant increase in insulin secretion during the 2^nd^ phase of the hyperglycemic clamp in NGT individuals [11].

### Mitochondrial ATP synthesis

**Fig 2:**
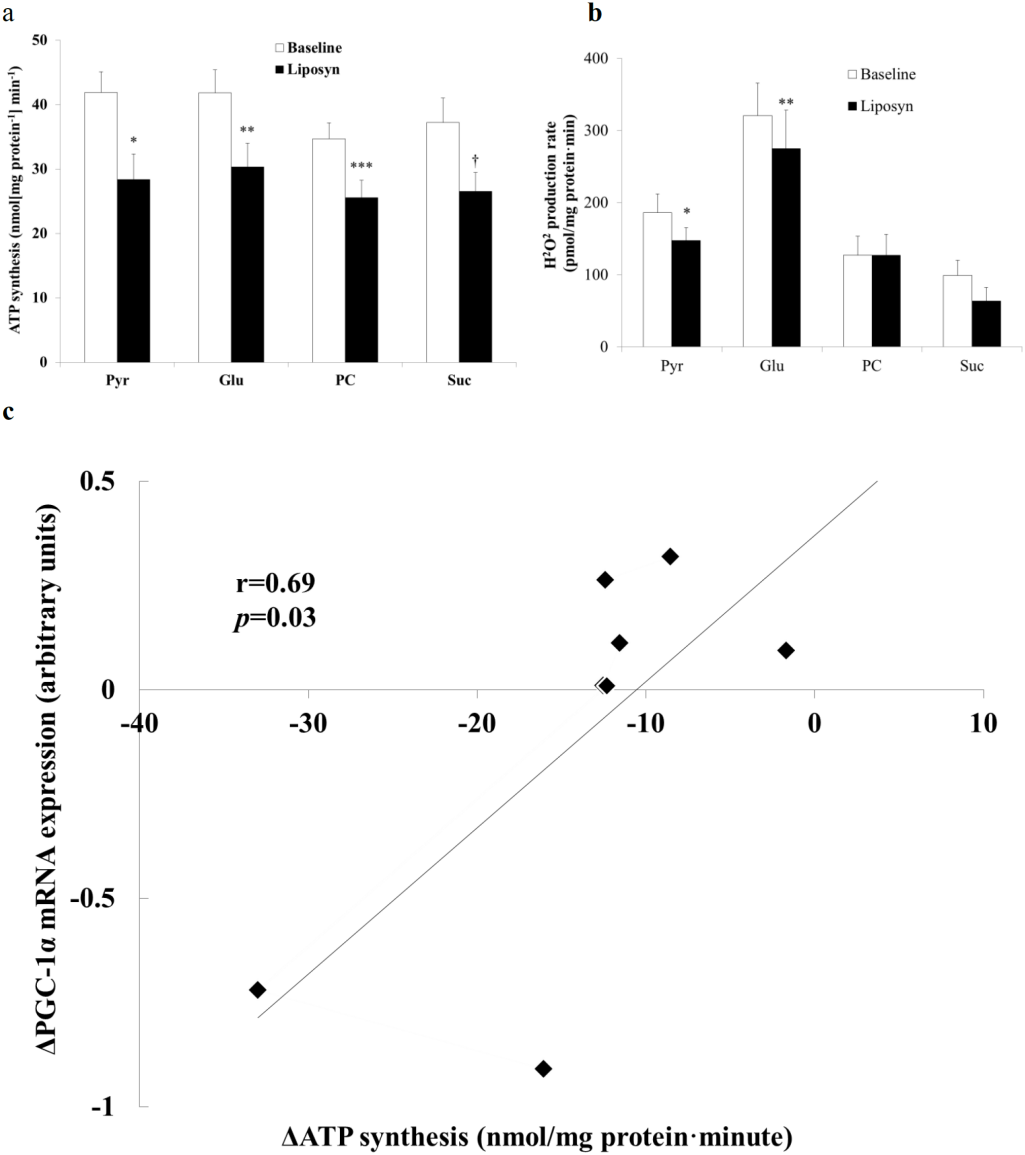
Effect of chronic (3 day) physiologic increase in plasma FFA concentration on mitochondrial function and PGC-1α mRNA expression. **a:** Rate of mitochondrial ATP production with complex I substrates 2.5 mM pyruvate (Pyr), 2.5 mM glutamate (Glu), 0.5 mM palmitoyl-L-carnitine (PC) and the complex II substrate 5 mM succinate plus 0.001 mM rotenone (Suc) (*p = 0.002, **p = 0.004, ***p = 0.001, †*p* = 0.005 paired student’s t-test). **b**: Rate of mitochondrial hydrogen peroxide production with complex I substrates 2.5 mM pyruvate (Pyr), 2.5 mM glutamate (Glu), 0.5 mM palmitoyl-L-carnitine (PC) and the complex II substrate 5 mM succinate plus 0.001 mM rotenone (Suc) (*p = 0.02, **p = 0.03, paired student’s t-test). **c:** Correlation between change in individual subject mitochondrial complex I function (ΔATP synthesis with pyruvate) and change in individual subject PGC-1α mRNA expression (Δ PGC-1α mRNA expression) following lipid infusion. Results are from 8 subjects with available data. PGC-1α mRNA expression was assessed by real time rtPCR. PGC-1α levels were corrected for HMBS gene expression. Complex I function was measured by ATP synthesis with pyruvate. *p* = 0.03 one-tailed Pearsons correlation.

Due to technical problems, mitochondrial ATP synthesis was not tested in 1 of the 9 subjects and ATP synthesis data are presented on 8 subjects. FFA-induced insulin resistance was accompanied by reduced mitochondrial ATP synthesis with complex 1 (by ~ 34±9%, *p*<0.05) and complex 2 (by 30±5%, *p*<0.05) substrates (Fig 2a). When results obtained from men and women were analyzed separately the trend towards reduced mitochondrial function did not change. Mitochondrial ROS generation was significantly lower with pyruvate and glutamate, but not with palmitoyl-L-carnitine or succinate (Fig 2b).

### PGC-1α expression

Mean skeletal muscle PGC-1α mRNA expression of the complete cohort did not change significantly following Liposyn infusion (0.83±0.08 *vs*. 0.73±0.10) (n = 9). However, the change in PGC-1α mRNA expression levels in individual subjects correlated positively (r = 0.69, *p* = 0.03, one-tailed Pearsons correlation) with the change in pyruvate-induced mitochondrial ATP synthesis following lipid infusion (Fig 2c). Higher levels of PGC-1α mRNA expression were associated with a smaller reduction in ATP synthesis in response to increased lipid availability. These observations align with previous publications suggesting a decline in mitochondrial ATP synthesis in response to lipid exposure [25]) and an association between PGC-1α expression and mitochondrial ATP synthesis [27–29].

### PGC-1α promoter methylation

**Fig 3:**
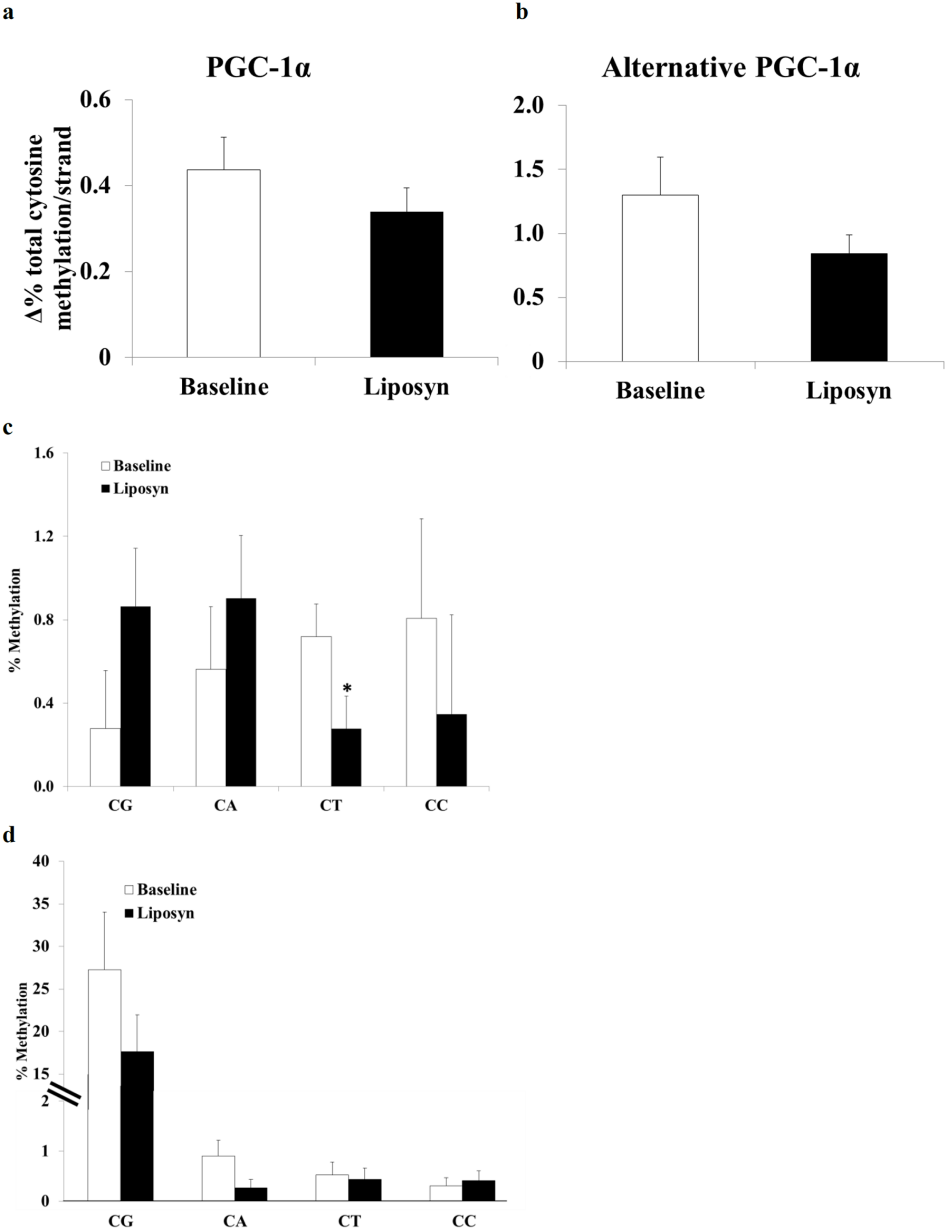
Effect of chronic (3 day) physiologic increase in plasma FFA concentration on PGC-1α promoter methylation. a: Change in % methylation/strand of all cytosine residues in PGC-1α (left) and b: alternative PGC-1α (right) promoters (n = 9). Alternative PGC-1α *p* = 0.055 vs baseline study without Liposyn infusion. c,d: Change in % methylation of each cytosine residue combination in the PGC-1α promoter before and after lipid infusion. c: % methylation of specific cytosine-residue combinations relative to total cytosine residues in the amplified strand (CG-4; CA-21; CT-29; CC-9) (n = 9). *p = 0.002 for CT methylation. d: % methylation of specific cytosine-residue combination relative to total cytosine residue combinations in the amplified strand (n = 9).

**Fig 4:**
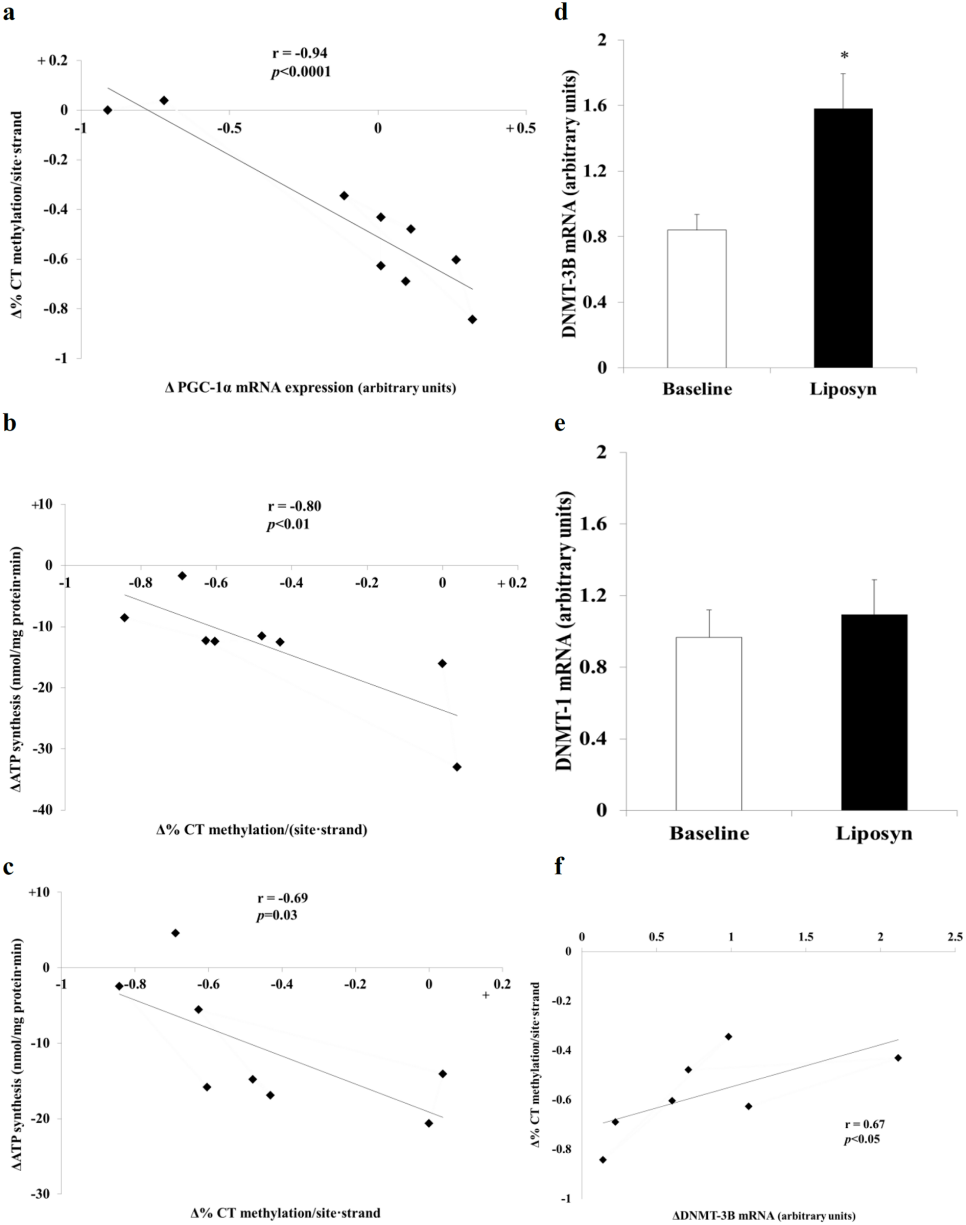
Correlation between the change in CpT methylation in the PGC-1α promoter, PGC-1α mRNA expression & mitochondrial function. **a:** Correlation between the change in CpT methylation in the PGC-1α promoter and PGC-1α mRNA expression following prolonged (3 day) lipid infusion (n = 9) (r = -0.94, *p*<0.0001). **b:** Correlation between the change in CpT methylation in the PGC-1α promoter and change in mitochondrial Complex I function following prolonged lipid infusion (n = 8). Complex I function was measured by ATP synthesis with pyruvate (r = -0.80, *p*<0.01). **c:** Correlation between the change in CpT methylation in the PGC-1α promoter and change in mitochondrial Complex II function following prolonged lipid infusion (n = 8). Complex II function was measured by ATP synthesis with succinate + the complex I inhibitor rotenone (r = -0.69, *p* = 0.03). **d, e:** Real-time PCR analysis of DNMT-3B and DNMT-1 change in expression after lipid infusion. RNA was extracted from vastus lateralis muscle biopsies obtained at baseline and after lipid infusion for 72 hours. Results are shown as fold induction normalized to HMBS values. *p = 0.005 for change in DNMT-3B expression level between baseline and Liposyn exposure. *p* = 0.27 for change in DNMT-1 expression level between baseline and Liposyn exposure. n = 9. **f**: Correlation between the change in CpT methylation in the PGC-1α promoter and DNMT-3B mRNA expression following prolonged lipid infusion amongst a subset of “responders” (7 of 9 subjects excluding two subjects who had minimal or no CpT demethylation), (n = 7). DNMT-3B mRNA expression was assessed by real time rtPCR and corrected for HMBS gene expression (r = 0.67, *p*<0.05).

Lipid infusion had no effect on total PGC-1α promoter methylation or on non CpG methylation, while there was a non-statistically significant trend towards reduced methylation of the alternative PGC-1α promoter (0.84±0.15% *vs*. 1.29±0.29% methylated residues/patient•strand, *p* = 0.055) (Fig 3a and 3b). When the following specific cytosine residue combinations (CpG, CpC, CpT, CpA) in PGC-1α promoter were examined, a significant decrease in CpT methylation was demonstrated (0.28±0.09% *vs*. 0.72±0.16% methylated residues/subject, *p* = 0.002) (Fig 3c and S3 Fig)] with no change in CpT methylation in Alt- PGC-1α promoter (Fig 3d). The decrease in CpT methylation was observed both in men and women when each group was analyzed separately. Reduction in CpT methylation was inversely correlated with the individual change in PGC-1α mRNA expression (r = -0.94, p<0.0001) (Fig 4a), suggesting an inhibitory effect of CpT methylation on PGC-1α mRNA expression. A similar trend with high r values was obtained when samples from men and women were analyzed separately (r = 0.946 men and r = 0.939 in women). Similarly, change in CpT methylation correlated negatively with lipid-associated reduction in ATP synthesis by mitochondrial complex 1 activity (ATP synthesis with pyruvate; r = -0.80, *p*<0.01) (Fig 4b) and complex 2 activity (ATP synthesis with succinate plus rotenone; r = -0.69; *p* = 0.03) (Fig 4c). Thus, CpT de-methylation during prolonged FFA exposure was associated with preservation of mitochondrial ATP synthesis. It is notable that the two subjects with minimal or no change in CpT methylation are the subjects with the greatest change in PGC-1α mRNA expression and the greatest decline in mitochondrial complex 1 activity. While these individuals may be considered outliers, their response during the insulin clamp studies, their plasma FFA levels, and other physiologic parameters did not differ from the rest of the cohort. Because of the consistent response with respect to methylation, gene expression and mitochondrial function assays, we believe that the results in these 2 subjects represent a physiologic response to lipid exposure. Correlation beteween CpT methylation and mitochondrial H^2^O^2^ production was less consistent; while a significant negative correlation was noted with the complex 1 substrates glutamate (r = -0.66, *p* = 0.038) and palmitoyl-L-carnitine (r = -0.865, *p* = 0.003), no correlation was observed with pyruvate and the complex 2 substrate succinate (S4 Fig).

In mammals, three DNA methyltransferases have been identified: DNMT-1 is considered to be a maintenance DNA methyltransferase, while DNMT-3A and DNMT-3B contribute to *de novo* DNA methylation [30]. In skeletal muscle from NGT and T2DM subjects, quantitative analysis of DNMT isoforms using RT-PCR revealed an increase in mRNA expression of DNMT3B in T2DM versus NGT subjects while DNMT1 and DNMT3A did not change. Consistent with this, we measured DNMT-3A in 5 subjects (corrected for 18S) and failed to observe any change from baseline (0.83 ± 0.43, *p* = 0.19 arbitrary PCR units). Liposyn infusion resulted in significant increase in DNMT-3B expression (1.61 ± 0.22 to 0.87 ± 0.09 arbitrary PCR units relative to HMBS expression, *p* = 0.005) without change in DNMT-1 expression (1.09 ± 0.19 *vs*. 0.96 ± 0.15 arbitrary PCR units relative to HMBS expression, *p* = NS) (Fig 4d and 4e). In an *ex vivo* human skeletal muscle model, DNMT-3B silencing with siRNA prevented palmitate-induced down regulation of PGC-1α mRNA expression [20]. Amongst a subset of “responders” (7 of 9 subjects, excluding the two subjects mentioned above who had minimal or no change in CpT methylation), a greater increase in individual DNMT-3B expression correlated with less Liposyn-induced CpG demethylation (i.e. demethylation of the PGC-1α promoter was lower in subjects in whom DNMT3B mRNA expression was higher) (Fig 4f) (*p* = 0.04, r = 0.67). A non-statistically significant inverse trend was noted between DNMT-3B expression and change in individual PGC-1α expression level (*p* = 0.07, r = 0.60).

Lastly, we combined all values obtained at baseline and during Liposyn treatment, looking for correlation between absolute levels of methylation, PGC-1α and DNMT-3B mRNA expression levels and mitochondrial ATP production with complex 1 and complex 2 substrates. %CT methylation/site strand was significantly and negatively correlated with DNMT-3B mRNA expression (S4e Fig r = -0.48, p = 0.02) and ATP production with complex 1 substrates was correlated with ATP production with complex 2 substrates (r = 0.902, *p*<0.001)

## Discussion

The lipotoxic effect of elevated plasma FFA and intracellular lipid metabolites (FACoAs, diacylglycerol, ceramides) on insulin sensitivity in muscle/liver and beta cell function is well established (2–4). We previously demonstrated that increased lipid availability impairs insulin signaling and decreases insulin-mediated glucose disposal in lean NGT individuals [22]. Conversely, reduced lipid availability improves insulin-mediated glucose disposal in NGT obese and T2DM individuals [12, 13, 31, 32]. The present results confirm these observations by demonstrating reduced peripheral and hepatic insulin sensitivity (Fig 1a and 1b) in response to chronic (3 day) lipid infusion and extends these results by demonstrating that a physiologic increase in plasma FFA reduces skeletal muscle mitochondrial ATP synthesis.

PGC-1α plays a central role in muscle mitochondrial biogenesis and function in response to external stimuli [18]. A possible mechanism via which PGC-1α mediates this interaction is through regulation of PGC-1α expression via modification of PGC-1α promoter methylation. In healthy subjects, acute exercise causes demethylaton of PGC-1α promoter in skeletal muscle, resulting in dose-dependent increase in PGC-1α mRNA expression [33]. Conversely, increased PGC-1α promoter methylation was observed in skeletal muscle of diabetic subjects and in myotubes exposed to palmitate *ex vivo*. Of note, the increase in methylation occurred primarily in non-CpG residues and was associated with an increase in DNMT-3B expression [20].

Therefore, we examined the *in vivo* effect of a chronic (72-hour) physiologic increase in lipid availability on PGC-1α promoter methylation in NGT subjects. Despite lack of effect on total PGC-1α methylation or CpG residue methylation, we observed a significant reduction in CpT residue methylation, which correlated with both PGC-1α mRNA expression levels (Fig 4a) and parameters of mitochondrial oxidative function (Fig 4b and 4c). DNMT-3B levels increased after lipid infusion and were correlated with preserved methylation of CpT residues. Thus, subjects with greater CpT demethylation in response to lipid infusion had greater expression of PGC-1α mRNA and relatively preserved mitochondrial function. In contrast, subjects with a greater increase in DNMT-3B levels following lipid infusion manifested reduced CpT demethylation (Fig 4f). A similar and negative correlation between absolute values of %CT methylation/site strand and DNMT-3B mRNA expression levels was observed (this analysis was conducted on absolute, combined baseline and Liposyn values).

The alternative PGC-1α promoter recently has been identified approximately 13.7kb upstream of originally identified PGC-1α promoter and yields alternative transcripts which are present at much lower levels [34]. Interestingly, methylation of the alternative PGC-1α promoter was similar between healthy individuals and individuals with T2DM and expression of it’s specific isoform, despite rapidly increasing after exercise was not associated with changes in DNA methylation in a mouse skeletal muscle [20, 35, 36]. In our study, a non-statistically significant trend towards reduced methylation was noted in the alternative PGC-1α promoter and no change in individual residue methylation levels was observed.

These results suggest two opposing responses to increased lipid availability in healthy NGT subjects:

1. a previously undescribed ‘protective’ mechanism via which elevated plasma FFA/intramyocellular lipids cause PGC-1α promoter CpT demethylation, preserved PGC-1α mRNA expression and preserved mitochondrial function. The enzymatic process leading to demethylation is poorly defined but may involve Tet catalyzed oxidation followed by decarboxylation of methylcytosine [37] or activation of cytidine deaminase or apolipoprotein B mRNA editing enzyme 1 [38].
2. The above sequence is opposed by an increase in DNMT-3B expression, which preserves (increases) CpT methylation and decreases PGC-1α mRNA expression. This is supported by the observation that absolute levels of DNMT-3B were higher when absolute %methylation levels of the PGC-1α promoter were lower. The strong correlative relationships between these opposing processes support this hypothesis. We hypothesize that the insulin resistant, obese diabetic phenotype emerges when the DNMT-3B-mediated increase in PGC-1α promoter methylation and subsequent mitochondrial dysfunction outweighs the beneficial effect of PGC-1α promoter demethylation and preservation of mitochondrial function.

Environmental-induced changes in non-CpG methylation in mammals have been described in human and murine mitochondrial DNA control regions (D-loop) [39] and in adult murine brains in regions with low CpG density [40]. The later is similar to the area we examined in the PGC-1α promoter. Functionally, non-CpG methylation is responsive to environmental changes and exerts an inhibitory effect on gene expression [40]. Thus, myotubes exposed *ex vivo* to palmitate for 48-hours demonstrate increase PGC-1α promoter mRNA, non-CpG methylation and PGC-1α expression [20]. Conversely, total non-CpG methylation declined in murine aortic genomic DNA after prolonged diet-induced hyperlipidemia [41]. In our study we demonstrated significant changes in CpT methylation without overall effect on total methylation. This may be explained, in part, by the relative abundance of CpT sites in the PGC-1α promoter relative to other potential methylation sites (29 CpT sites *vs*. 4 CpG sites; 21 CpA sites and 9 CpC sites) by an as-of-yet undefined CpT specific process, by species differences, i.e. human vs murine, or by the intervention, i.e. lipid infusion.

The present study has several limitations including the relatively small number of subjects, variability between subjects, the lack of control group and the “short” exposure time to increased lipid availability. With respect to the later, the obese T2DM phenotype develops after many years of excess caloric intake. However, with this limitation in mind, our study provides insight into the very early stages of the interaction between excess lipid intake and mitochondrial DNA methylation in development of the obese diabetic phenotype at an early pathophysiologic stage, before overt lipid-associated insulin resistance emerges. As such, these observations provide novel insights that may hold the key to preventing and overcoming the rampant diabetes/obesity pandemic.

## Supporting information

S1 Fig Hyperinsulinemic euglycemic clamp data. **a:** pre-clamp fasting insulin levels. p<0.001 n = 9. **b:** Time course plot of plasma insulin concentrations during the hyperinsulinemic euglycemic clamp. **c**: Time course plot of plasma glucose concentrations during the hyperinsulinemic euglycemic clamp. **d**: Basal hepatic glucose production at baseline and after Liposyn treatment.
(TIFF)

S2 Fig Two-step hyperglycemic clamp (+125 and +300 mg/dL) with arginine data. **a:** Time course plot of plasma glucose concnetrations. **b-d:** Time course plot of plasma insulin concnetrations during the 1st, 2nd and arginine steps of the clamp.
(TIFF)

S3 Fig Individual subject change from baseline in %CT methylation/site strand in the PGC-1α promoter after Liposyn exposure. (TIFF)

S4 Fig Correlation between the change in CpT methylation in the PGC-1α promoter, and mitochondrial H^2^O^2^ production function. **a-d:** Correlation between the change in CpT methylation in the PGC-1α promoter and change in mitochondrial Complex I and complex II H^2^O^2^ production following prolonged lipid infusion (n = 8). **e:** Correlation between absolute DNMT-3B mRNA expression levels and absolute %CT methylation/site strand in the PGC-1α promoter (data was combined from baseline and post Liposyn exposure)
(TIFF)

## References

[1] Defronzo RA. Banting Lecture. From the triumvirate to the ominous octet: a new paradigm for the treatment of type 2 diabetes mellitus. Diabetes. 2009;58(4):773–95. doi: 10.2337/db09-9028 . 19336687 10.2337/db09-9028 PMC2661582

[2] Unger RH. Lipotoxicity in the pathogenesis of obesity-dependent NIDDM. Genetic and clinical implications. Diabetes. 1995;44(8):863–70. . 7621989 10.2337/diab.44.8.863

[3] Prentki M, Nolan CJ. Islet beta cell failure in type 2 diabetes. J Clin Invest. 2006;116(7):1802–12. doi: 10.1172/JCI29103 . 16823478 10.1172/JCI29103 PMC1483155

[4] Bays H, Mandarino L, DeFronzo RA. Role of the adipocyte, free fatty acids, and ectopic fat in pathogenesis of type 2 diabetes mellitus: peroxisomal proliferator-activated receptor agonists provide a rational therapeutic approach. J Clin Endocrinol Metab. 2004;89(2):463–78. doi: 10.1210/jc.2003-030723 . 14764748 10.1210/jc.2003-030723

[5] Bonadonna RC, Zych K, Boni C, Ferrannini E, DeFronzo RA. Time dependence of the interaction between lipid and glucose in humans. Am J Physiol. 1989;257(1 Pt 1):E49–56. . 2665518 10.1152/ajpendo.1989.257.1.E49

[6] Ferrannini E, Barrett EJ, Bevilacqua S, DeFronzo RA. Effect of fatty acids on glucose production and utilization in man. J Clin Invest. 1983;72(5):1737–47. doi: 10.1172/JCI111133 . 6138367 10.1172/JCI111133 PMC370462

[7] Roden M, Krssak M, Stingl H, Gruber S, Hofer A, Furnsinn C, et al Rapid impairment of skeletal muscle glucose transport/phosphorylation by free fatty acids in humans. Diabetes. 1999;48(2):358–64. . 10334314 10.2337/diabetes.48.2.358

[8] Boden G, Jadali F, White J, Liang Y, Mozzoli M, Chen X, et al Effects of fat on insulin-stimulated carbohydrate metabolism in normal men. J Clin Invest. 1991;88(3):960–6. doi: 10.1172/JCI115399 . 1885781 10.1172/JCI115399 PMC295496

[9] Bajaj M, Pratipanawatr T, Berria R, Pratipanawatr W, Kashyap S, Cusi K, et al Free fatty acids reduce splanchnic and peripheral glucose uptake in patients with type 2 diabetes. Diabetes. 2002;51(10):3043–8. . 12351445 10.2337/diabetes.51.10.3043

[10] Kashyap SR, Belfort R, Berria R, Suraamornkul S, Pratipranawatr T, Finlayson J, et al Discordant effects of a chronic physiological increase in plasma FFA on insulin signaling in healthy subjects with or without a family history of type 2 diabetes. Am J Physiol Endocrinol Metab. 2004;287(3):E537–46. doi: 10.1152/ajpendo.00541.2003 . 15126243 10.1152/ajpendo.00541.2003

[11] Kashyap S, Belfort R, Gastaldelli A, Pratipanawatr T, Berria R, Pratipanawatr W, et al A sustained increase in plasma free fatty acids impairs insulin secretion in nondiabetic subjects genetically predisposed to develop type 2 diabetes. Diabetes. 2003;52(10):2461–74. . 14514628 10.2337/diabetes.52.10.2461

[12] Bajaj M, Suraamornkul S, Kashyap S, Cusi K, Mandarino L, DeFronzo RA. Sustained reduction in plasma free fatty acid concentration improves insulin action without altering plasma adipocytokine levels in subjects with strong family history of type 2 diabetes. J Clin Endocrinol Metab. 2004;89(9):4649–55. doi: 10.1210/jc.2004-0224 . 15356076 10.1210/jc.2004-0224

[13] Daniele G, Eldor R, Merovci A, Clarke GD, Xiong J, Tripathy D, et al Chronic reduction of plasma free fatty acid improves mitochondrial function and whole-body insulin sensitivity in obese and type 2 diabetic individuals. Diabetes. 2014;63(8):2812–20. doi: 10.2337/db13-1130 . 24353180 10.2337/db13-1130 PMC4113069

[14] Petersen KF, Dufour S, Befroy D, Garcia R, Shulman GI. Impaired mitochondrial activity in the insulin-resistant offspring of patients with type 2 diabetes. N Engl J Med. 2004;350(7):664–71. doi: 10.1056/NEJMoa031314 . 14960743 10.1056/NEJMoa031314 PMC2995502

[15] Abdul-Ghani MA, Jani R, Chavez A, Molina-Carrion M, Tripathy D, Defronzo RA. Mitochondrial reactive oxygen species generation in obese non-diabetic and type 2 diabetic participants. Diabetologia. 2009;52(4):574–82. doi: 10.1007/s00125-009-1264-4 . 19183935 10.1007/s00125-009-1264-4

[16] Kelley DE, He J, Menshikova EV, Ritov VB. Dysfunction of mitochondria in human skeletal muscle in type 2 diabetes. Diabetes. 2002;51(10):2944–50. . 12351431 10.2337/diabetes.51.10.2944

[17] Fernandez-Marcos PJ, Auwerx J. Regulation of PGC-1alpha, a nodal regulator of mitochondrial biogenesis. Am J Clin Nutr. 2011;93(4):884S–90. doi: 10.3945/ajcn.110.001917 . 21289221 10.3945/ajcn.110.001917 PMC3057551

[18] Wu Z, Puigserver P, Andersson U, Zhang C, Adelmant G, Mootha V, et al Mechanisms controlling mitochondrial biogenesis and respiration through the thermogenic coactivator PGC-1. Cell. 1999;98(1):115–24. doi: 10.1016/S0092-8674(00)80611-X . 10412986 10.1016/S0092-8674(00)80611-X

[19] Richardson DK, Kashyap S, Bajaj M, Cusi K, Mandarino SJ, Finlayson J, et al Lipid infusion decreases the expression of nuclear encoded mitochondrial genes and increases the expression of extracellular matrix genes in human skeletal muscle. J Biol Chem. 2005;280(11):10290–7. doi: 10.1074/jbc.M408985200 . 15598661 10.1074/jbc.M408985200

[20] Barres R, Osler ME, Yan J, Rune A, Fritz T, Caidahl K, et al Non-CpG methylation of the PGC-1alpha promoter through DNMT3B controls mitochondrial density. Cell Metab. 2009;10(3):189–98. doi: 10.1016/j.cmet.2009.07.011 . 19723495 10.1016/j.cmet.2009.07.011

[21] Brons C, Jacobsen S, Nilsson E, Ronn T, Jensen CB, Storgaard H, et al Deoxyribonucleic acid methylation and gene expression of PPARGC1A in human muscle is influenced by high-fat overfeeding in a birth-weight-dependent manner. J Clin Endocrinol Metab. 2010;95(6):3048–56. doi: 10.1210/jc.2009-2413 . 20410232 10.1210/jc.2009-2413

[22] Chavez AO, Kamath S, Jani R, Sharma LK, Monroy A, Abdul-Ghani MA, et al Effect of short-term free Fatty acids elevation on mitochondrial function in skeletal muscle of healthy individuals. J Clin Endocrinol Metab. 2010;95(1):422–9. doi: 10.1210/jc.2009-1387 . 19864449 10.1210/jc.2009-1387 PMC2805487

[23] DeFronzo RA, Tobin JD, Andres R. Glucose clamp technique: a method for quantifying insulin secretion and resistance. Am J Physiol. 1979;237(3):E214–23. . 382871 10.1152/ajpendo.1979.237.3.E214

[24] Cusi K, Maezono K, Osman A, Pendergrass M, Patti ME, Pratipanawatr T, et al Insulin resistance differentially affects the PI 3-kinase- and MAP kinase-mediated signaling in human muscle. J Clin Invest. 2000;105(3):311–20. doi: 10.1172/JCI7535 . 10675357 10.1172/JCI7535 PMC377440

[25] Abdul-Ghani MA, Muller FL, Liu Y, Chavez AO, Balas B, Zuo P, et al Deleterious action of FA metabolites on ATP synthesis: possible link between lipotoxicity, mitochondrial dysfunction, and insulin resistance. Am J Physiol Endocrinol Metab. 2008;295(3):E678–85. doi: 10.1152/ajpendo.90287.2008 . 18593850 10.1152/ajpendo.90287.2008

[26] Steele R, Wall JS, De Bodo RC, Altszuler N. Measurement of size and turnover rate of body glucose pool by the isotope dilution method. Am J Physiol. 1956;187(1):15–24. . 13362583 10.1152/ajplegacy.1956.187.1.15

[27] Arany Z, He H, Lin J, Hoyer K, Handschin C, Toka O, et al Transcriptional coactivator PGC-1 alpha controls the energy state and contractile function of cardiac muscle. Cell Metab. 2005;1(4):259–71. doi: 10.1016/j.cmet.2005.03.002 . 16054070 10.1016/j.cmet.2005.03.002

[28] Lehman JJ, Boudina S, Banke NH, Sambandam N, Han X, Young DM, et al The transcriptional coactivator PGC-1alpha is essential for maximal and efficient cardiac mitochondrial fatty acid oxidation and lipid homeostasis. Am J Physiol Heart Circ Physiol. 2008;295(1):H185–96. doi: 10.1152/ajpheart.00081.2008 . 18487436 10.1152/ajpheart.00081.2008 PMC2494758

[29] Choi CS, Befroy DE, Codella R, Kim S, Reznick RM, Hwang YJ, et al Paradoxical effects of increased expression of PGC-1alpha on muscle mitochondrial function and insulin-stimulated muscle glucose metabolism. Proc Natl Acad Sci U S A. 2008;105(50):19926–31. doi: 10.1073/pnas.0810339105 . 19066218 10.1073/pnas.0810339105 PMC2598730

[30] Okano M, Bell DW, Haber DA, Li E. DNA methyltransferases Dnmt3a and Dnmt3b are essential for de novo methylation and mammalian development. Cell. 1999;99(3):247–57. . 10555141 10.1016/s0092-8674(00)81656-6

[31] Liang H, Tantiwong P, Sriwijitkamol A, Shanmugasundaram K, Mohan S, Espinoza S, et al Effect of a sustained reduction in plasma free fatty acid concentration on insulin signalling and inflammation in skeletal muscle from human subjects. J Physiol. 2013;591(11):2897–909. doi: 10.1113/jphysiol.2012.247510 . 23529132 10.1113/jphysiol.2012.247510 PMC3690693

[32] Bajaj M, Medina-Navarro R, Suraamornkul S, Meyer C, DeFronzo RA, Mandarino LJ. Paradoxical changes in muscle gene expression in insulin-resistant subjects after sustained reduction in plasma free fatty acid concentration. Diabetes. 2007;56(3):743–52. doi: 10.2337/db06-0840 . 17327445 10.2337/db06-0840

[33] Barres R, Yan J, Egan B, Treebak JT, Rasmussen M, Fritz T, et al Acute exercise remodels promoter methylation in human skeletal muscle. Cell Metab. 2012;15(3):405–11. doi: 10.1016/j.cmet.2012.01.001 . 22405075 10.1016/j.cmet.2012.01.001

[34] Yoshioka T, Inagaki K, Noguchi T, Sakai M, Ogawa W, Hosooka T, et al Identification and characterization of an alternative promoter of the human PGC-1alpha gene. Biochem Biophys Res Commun. 2009;381(4):537–43. doi: 10.1016/j.bbrc.2009.02.077 . 19233136 10.1016/j.bbrc.2009.02.077

[35] Miura S, Kai Y, Kamei Y, Ezaki O. Isoform-specific increases in murine skeletal muscle peroxisome proliferator-activated receptor-gamma coactivator-1alpha (PGC-1alpha) mRNA in response to beta2-adrenergic receptor activation and exercise. Endocrinology. 2008;149(9):4527–33. doi: 10.1210/en.2008-0466 . 18511502 10.1210/en.2008-0466

[36] Lochmann TL, Thomas RR, Bennett JP Jr., Taylor SM. Epigenetic Modifications of the PGC-1alpha Promoter during Exercise Induced Expression in Mice. PLoS One. 2015;10(6):e0129647 doi: 10.1371/journal.pone.0129647 . 26053857 10.1371/journal.pone.0129647 PMC4460005

[37] Ito S, Shen L, Dai Q, Wu SC, Collins LB, Swenberg JA, et al Tet proteins can convert 5-methylcytosine to 5-formylcytosine and 5-carboxylcytosine. Science. 2011;333(6047):1300–3. doi: 10.1126/science.1210597 . 21778364 10.1126/science.1210597 PMC3495246

[38] Carey N, Marques CJ, Reik W. DNA demethylases: a new epigenetic frontier in drug discovery. Drug Discov Today. 2011;16(15–16):683–90. doi: 10.1016/j.drudis.2011.05.004 . 21601651 10.1016/j.drudis.2011.05.004

[39] Bellizzi D, D’Aquila P, Scafone T, Giordano M, Riso V, Riccio A, et al The control region of mitochondrial DNA shows an unusual CpG and non-CpG methylation pattern. DNA Res. 2013;20(6):537–47. doi: 10.1093/dnares/dst029 . 23804556 10.1093/dnares/dst029 PMC3859322

[40] Guo JU, Su Y, Shin JH, Shin J, Li H, Xie B, et al Distribution, recognition and regulation of non-CpG methylation in the adult mammalian brain. Nat Neurosci. 2014;17(2):215–22. doi: 10.1038/nn.3607 . 24362762 10.1038/nn.3607 PMC3970219

[41] Jiang Y, Zhang H, Sun T, Wang J, Sun W, Gong H, et al The comprehensive effects of hyperlipidemia and hyperhomocysteinemia on pathogenesis of atherosclerosis and DNA hypomethylation in ApoE-/- mice. Acta Biochim Biophys Sin (Shanghai). 2012;44(10):866–75. doi: 10.1093/abbs/gms075 . 23017835 10.1093/abbs/gms075

