# Supplementary figures and images for: Increased lipid availability for three days reduces whole body glucose uptake, impairs muscle mitochondrial function and initiates opposing effects on PGC-1α promoter methylation in healthy subjects
Source: PLoS One. 2017 Dec 20;12(12):e0188208. doi: 10.1371/journal.pone.0188208 (PMC5737973; doi:10.1371/journal.pone.0188208)

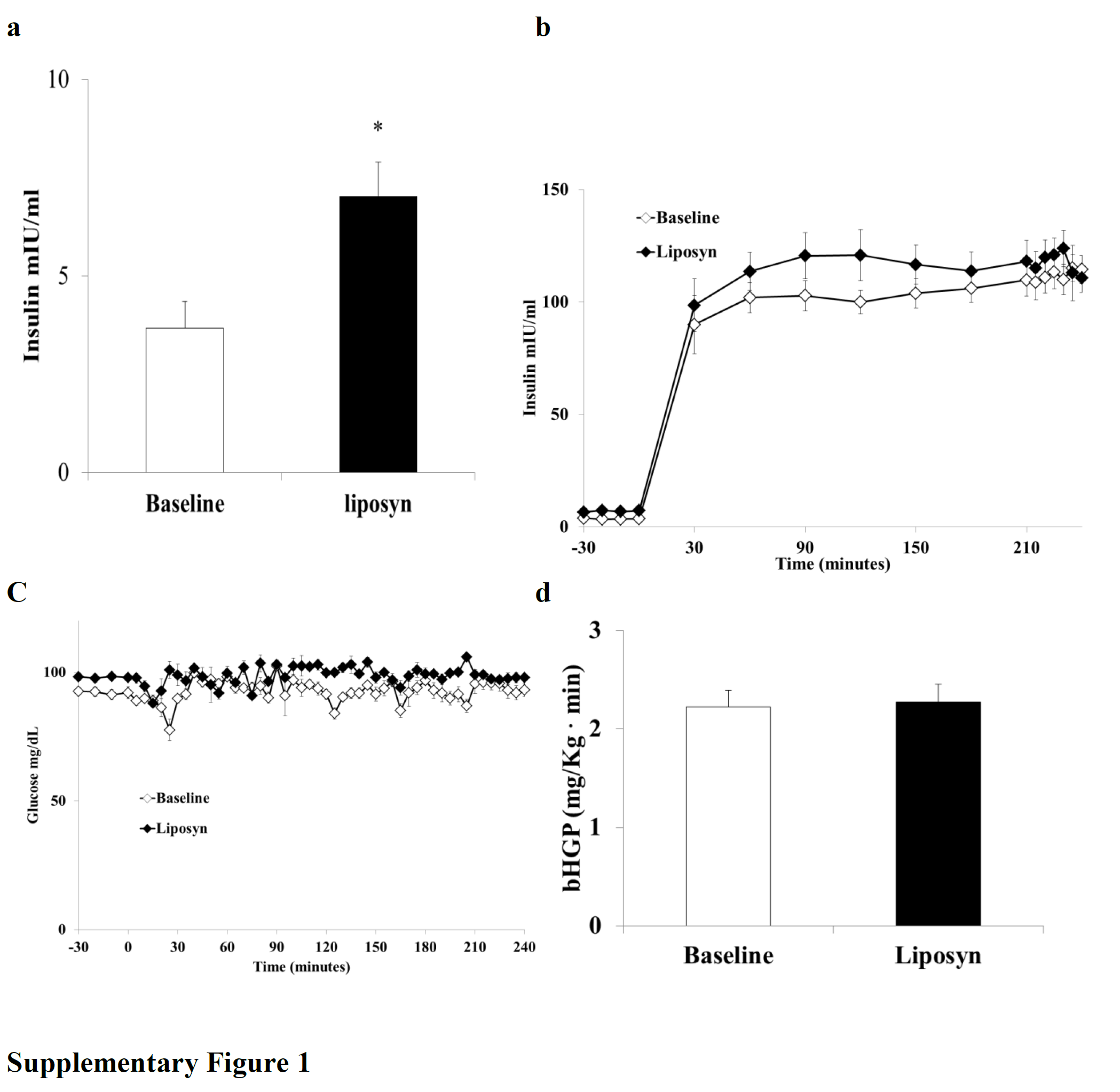

Supplement: S1 Fig — a: pre-clamp fasting insulin levels. p<0.001 n = 9. b: Time course plot of plasma insulin concentrations during the hyperinsulinemic euglycemic clamp. c: Time course plot of plasma glucose concentrations during the hyperinsulinemic euglycemic clamp. d: Basal hepatic glucose production at baseline and after Liposyn treatment. (TIFF) [file pone.0188208.s001.tiff]

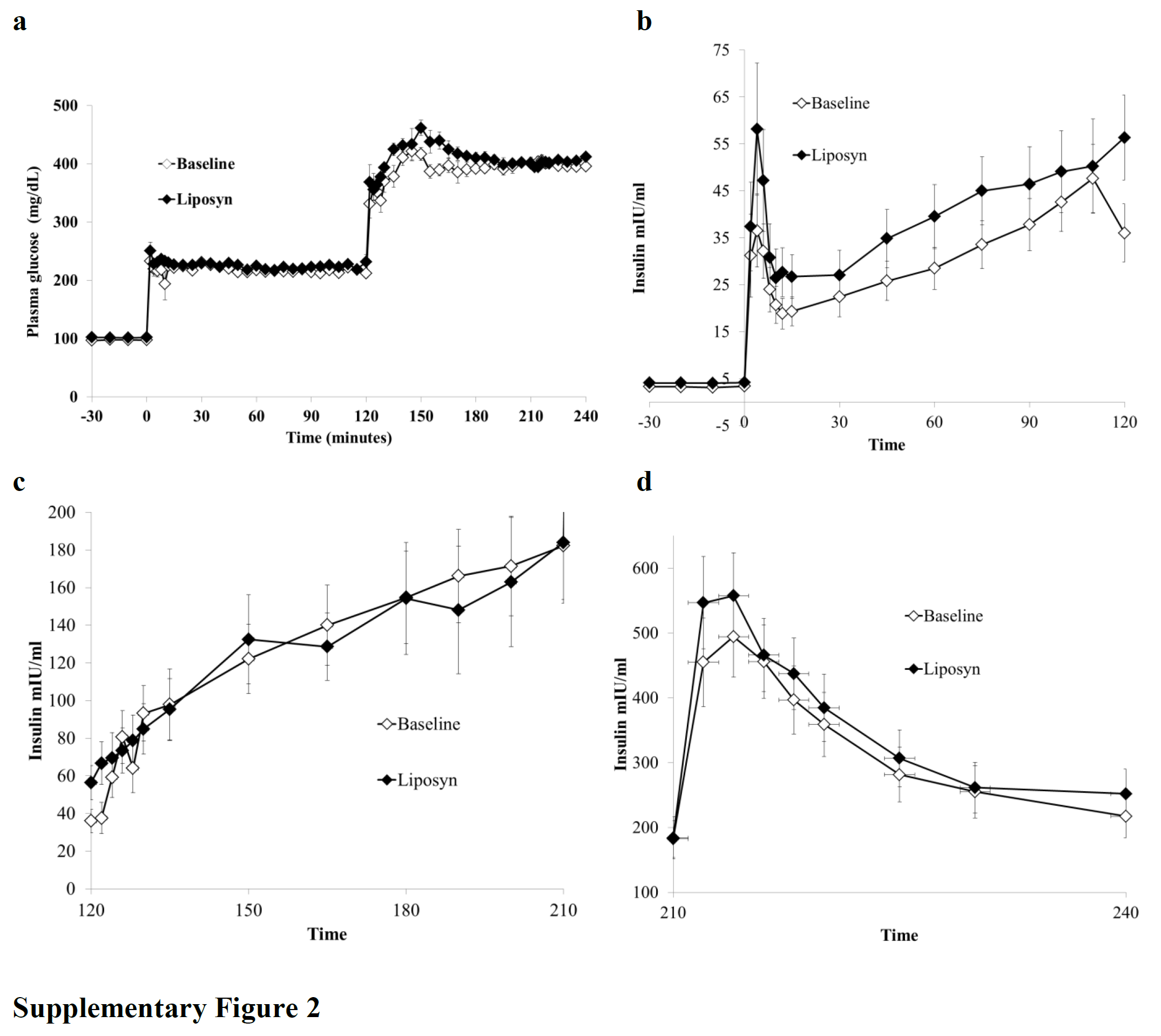

Supplement: S2 Fig — a: Time course plot of plasma glucose concnetrations. b-d: Time course plot of plasma insulin concnetrations during the 1st, 2nd and arginine steps of the clamp. (TIFF) [file pone.0188208.s002.tiff]

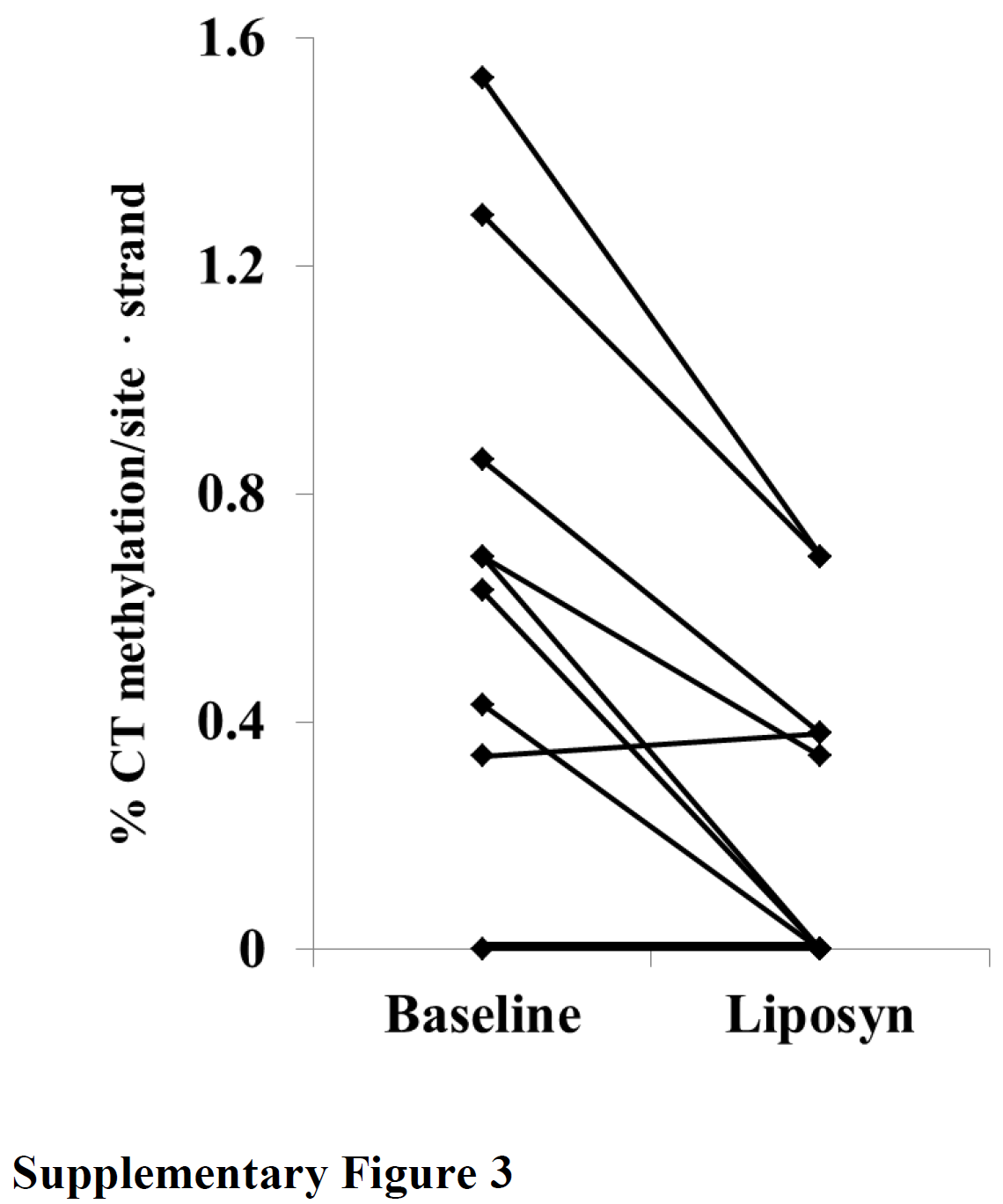

Supplement: S3 Fig — (TIFF) [file pone.0188208.s003.tiff]

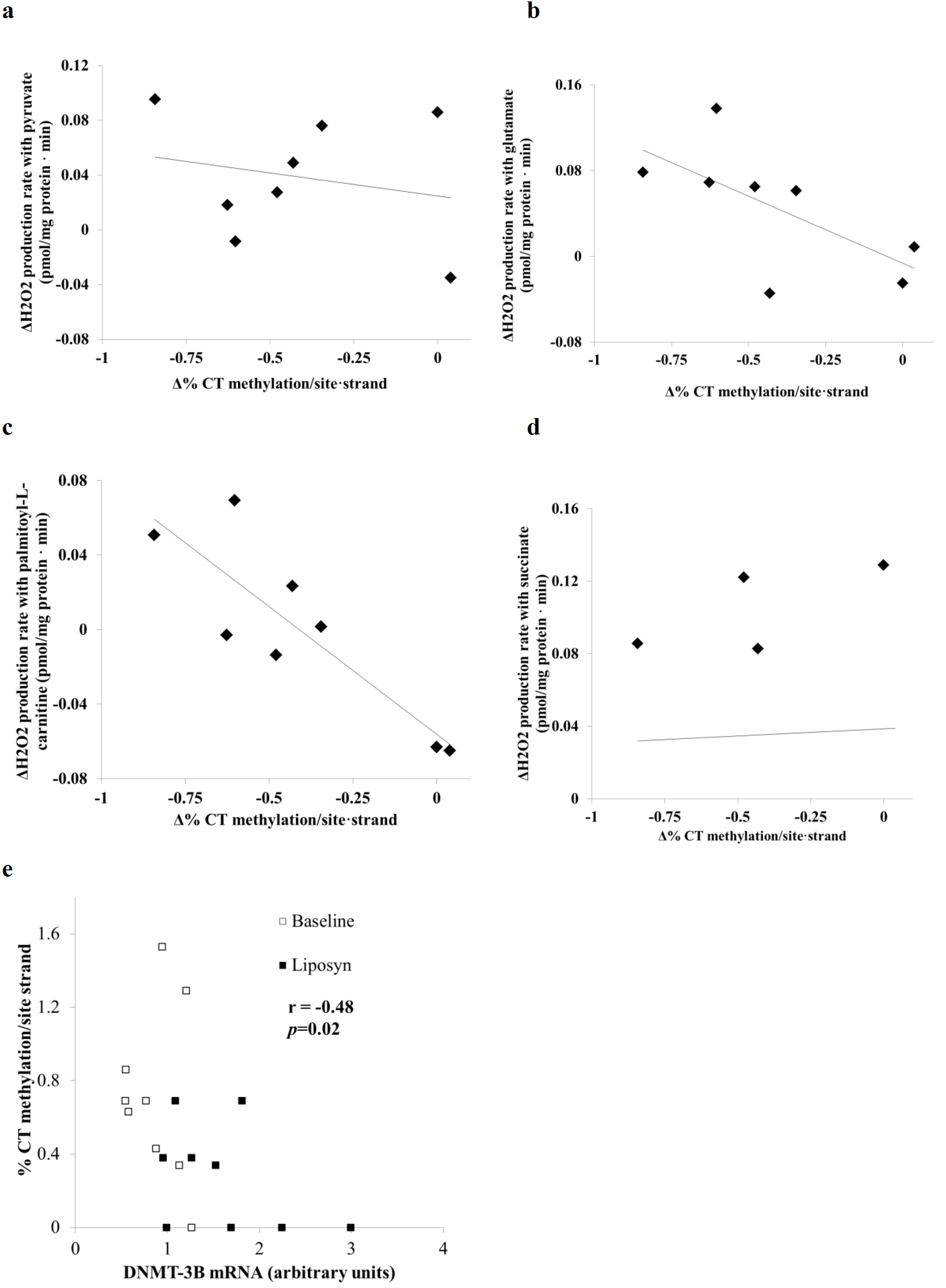

Supplement: S4 Fig — a-d: Correlation between the change in CpT methylation in the PGC-1α promoter and change in mitochondrial Complex I and complex II H2O2 production following prolonged lipid infusion (n = 8). e: Correlation between absolute DNMT-3B mRNA expression levels and absolute %CT methylation/site strand in the PGC-1α promoter (data was combined from baseline and post Liposyn exposure) (TIFF) [file pone.0188208.s004.tiff]
